# Multi-Faceted Functions of Secretory IgA at Mucosal Surfaces

**DOI:** 10.3389/fimmu.2013.00185

**Published:** 2013-07-12

**Authors:** Blaise Corthésy

**Affiliations:** ^1^R&D Laboratory, Department of Immunology and Allergy, University State Hospital Lausanne (CHUV), Lausanne, Switzerland

**Keywords:** secretory IgA, mucosal homeostasis, antibody, epithelium, infectious agents, commensal bacteria

## Abstract

Secretory IgA (SIgA) plays an important role in the protection and homeostatic regulation of intestinal, respiratory, and urogenital mucosal epithelia separating the outside environment from the inside of the body. This primary function of SIgA is referred to as immune exclusion, a process that limits the access of numerous microorganisms and mucosal antigens to these thin and vulnerable mucosal barriers. SIgA has been shown to be involved in avoiding opportunistic pathogens to enter and disseminate in the systemic compartment, as well as tightly controlling the necessary symbiotic relationship existing between commensals and the host. Clearance by peristalsis appears thus as one of the numerous mechanisms whereby SIgA fulfills its function at mucosal surfaces. Sampling of antigen-SIgA complexes by microfold (M) cells, intimate contact occurring with Peyer’s patch dendritic cells (DC), down-regulation of inflammatory processes, modulation of epithelial, and DC responsiveness are some of the recently identified processes to which the contribution of SIgA has been underscored. This review aims at presenting, with emphasis at the biochemical level, how the molecular complexity of SIgA can serve these multiple and non-redundant modes of action.

## Introduction

Secretory IgA (SIgA) is the principal immunoglobulin (Ig) on mucosal surfaces of humans and many other mammals. Globally, more IgA is produced than all other Ig isotypes combined. Due to its particular biosynthetic pathway relying on production by plasma cells in the lamina propria and poly Ig receptor (pIgR)-mediated secretion by epithelial cells overlying mucosal surfaces, SIgA displays a very different molecular form as compared to IgA antibodies found in the circulation and tissues. SIgA operates in an ever-changing environment whose function is to physically separate the inside of the body that needs to remain sterile from the outside world rich in antigenic stimuli including those present in air, liquid, and food. In the gastro-intestinal tract, a further challenge for host-defending SIgA is to discriminate between symbiotic harmless commensal bacteria and periodic invading, potentially life-threatening microorganisms. The complexity of mechanisms involved is far from being fully understood. From a more global immune surveillance’s point of view, the mucosal immune system, including SIgA, must constantly monitor the environment and maintain a balance between tolerance to the normal microbiota and immunity to microbial pathogens while the systemic immune system is designed to vigorously react to any foreign antigen or microbe. Given the intrinsic fragile nature of the gut and airway mucosal barriers ensured by a single layer of epithelial cells, the contribution of SIgA in maintaining homeostasis appears essential. This is reflected by the growing evidence of the role of maternal milk SIgA from early in life in the process of epithelial maturation. However, it is fair to mention that polymeric IgM actively transported across epithelia by pIgR (just like polymeric IgA), as well as IgG transuding from plasma into local secretions, can also participate in protection of the intestine and the respiratory tract (1–4).

**Figure 1:**
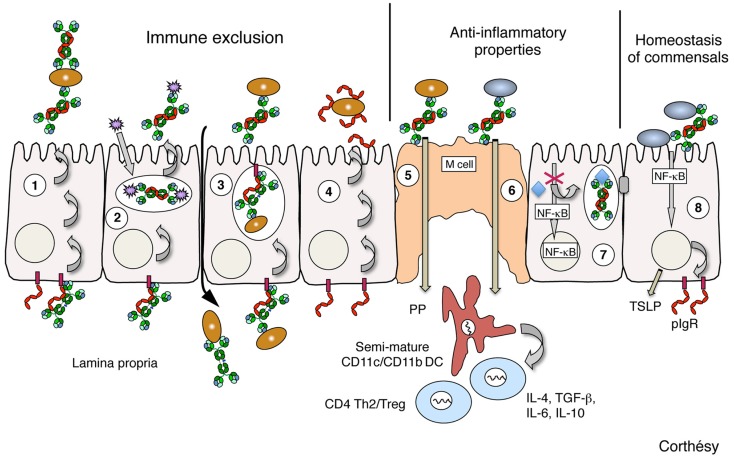
**Schematic representation of the identified levels by which polymeric IgA, SIgA, or SC may contribute to protection of mucosal surfaces, as defined in various *in vivo* and *in vitro* models**. (1) Polymeric IgA produced by local plasma cells in the lamina propria is transported across epithelial cells (a process referred to as transcytosis) by the polymeric Ig receptor (pIgR), and released in luminal secretions in the form of SIgA performing immune exclusion via interaction with environmental antigens (bacteria, viruses, toxins, etc). (2) Polymeric IgA on their way to pIgR-mediated secretions can intercept incoming viruses intracellularly, and excrete them in the form of non-virulent immune complexes. (3) Polymeric IgA may neutralize in the lamina propria invading infectious agents that have penetrated through breaches occurring in the inflamed epithelium; subsequent transport by pIgR will favor clearance of immune complexes. (4) Via glycans abundantly found on its surface, free SC released in secretions neutralizes pathogen-derived products, and contributes to protection of epithelial surfaces as well; this property is conserved when SC is bound to polymeric IgA in SIgA. (5) Sampling of SIgA by M cells in Peyer’s patches (PP) leads to specific targeting of the antibody to dendritic cells (DC) in the subepithelial dome region. In the form of immune complexes with noxious antigens, presentation to naïve T cells in the PP and draining mesenteric lymph nodes (not drawn) results in the onset of attenuated, Th2-biased mucosal immune responses with concomitant quenching of inflammatory circuits. (6) Remarkably, the same SIgA-mediated retro-transport is achieved with commensal bacteria, leading to the shaping of the mucosal immune system toward a non-inflammatory, tolerogenic pattern that takes place through the induction of regulatory T cells. (7) Neutralization of Gram-negative bacterial lipopolysaccharide (LPS) in apical recycling endosomes by transcytosing polymeric IgA abrogates NF-κB-mediated activation of pro-inflammatory gene products, thus preserving the epithelial barrier’s integrity. (8) Cross-talk between the probiotic bacteria and the intestinal mucosa is enhanced by SIgA, with various consequences extending from increased expression of epithelial pIgR and tight junction proteins to production of thymic stromal lymphopoietin (TSLP) involved in priming of mucosal DCs. Brown ellipses depict pathogen bacteria; gray ellipses depict commensal bacteria; purple spiky spheres depict virus; polymeric IgA are drawn in green; Free secretory component and polymeric Ig receptor (pIgR) are drawn in red; TSLP, thymic stromal lymphopoietin.

As this will become apparent when discussing the structure-function relationship, the various molecular forms of the antibody are highly glycosylated comprising sugar-derived residues in each constituent polypeptide. With respect to pIgA glycosylation, both human IgA1 and IgA2 subclasses have two conserved N-glycan sites on each heavy chain. Moreover, IgA2 preferentially found in secretions, harbors one or two additional N-glycans present on the Cα1 domain. IgA1 is the only subclass with O-carbohydrates in the hinge region. Mice have one class of IgA which is structurally similar to human IgA2 in terms of polypeptide assembly and glycosylation. In comparison with monomeric serum IgA, additional biochemical features found in SIgA include the joining (J) chain and secretory component (SC) (5), a polypeptide comprising the extracellular portion of the precursor pIgR that transports polymeric IgA across epithelial cells, a process also known as transcytosis (6) (Figure 1, pathway 1). The J chain, upon covalent binding to two IgA monomers, triggers dimerization (possibly, yet less commonly, oligomerization of higher magnitude) during biosynthesis in mucosal IgA-secreting plasma cells that are abundant in the lamina propria underlying the epithelium. With only one N-moiety, the J chain is the least glycosylated peptide constituent of SIgA. Incorporation of J chain within polymeric IgA (and IgM pentamers) is essential for selective recognition of the two antibody isotypes by membrane bound pIgR or purified free SC from colostrum or from recombinant origin. Carbohydrate residues represent up to 20% of the SC molecular mass, with seven sites of N-glycosylation identified (7). The function of SC in SIgA is manifold (see below), and may justify of why it is released in association with polymeric IgA from its precursor pIgR synthesized by epithelial cells after having ensured single transcytosis.

## Protective Operative Mechanisms Relevant to SIgA Function

Immune exclusion is the primary mechanism by which SIgA blocks microorganisms and toxins from attaching to mucosal target epithelial cells, thereby preventing surface damage, colonization, and subsequent massive invasion (8). In the context of the gut, immune exclusion is defined as the ability of SIgA, through its recognition of multiple antigenic epitopes on the surface of viruses and bacteria as well as proteins, to cross-link these various antigens in the intestinal lumen and consequently delay or abolish their intrinsic potential to adhere to and/or penetrate the epithelium (Figure 1, pathway 1). Such a consensual mode of action of SIgA against bacterial, viral, and parasitic mucosal pathogens, as well as toxins and possibly food allergens, has been defined via compelling evidence from animal models, *in vitro* models and human epidemiological studies.

IgA has been used in humans for passive protection or therapeutic intervention at mucosal surfaces (9–17), yet with different degrees of success, possibly because the complete SIgA molecule was not used. In the intestine of mice, passive oral delivery of specific IgA antibodies also protected against bacterial infections including *Salmonella typhimurium* (18, 19), *Vibrio cholera* (20), *Shigella flexneri* (21), and *Helicobacter pylori* (22). Monoclonal IgA antibodies directed against respiratory syncytial virus applied passively to the nasopharyngeal mucosa of mice subsequently prevented initial infection and pneumonia (23). Similar observations as to the crucial role of passively instilled IgA in preventing viral infection has been documented for influenza virus (24) and reovirus (25, 26). Intravenous injection of similar virus-neutralizing doses of anti-influenza polymeric IgA mAb, but not monomeric IgA, protected mice against viral infection due to transport into nasal secretions (27). Antigen-specific IgA antibodies produced by an IgA-secreting hybridoma clone implanted in the back of mice (backpack technique) were shown to provide efficient protection against *Vibrio cholerae* (28) and rotavirus (29) following pIgR-mediated transport into secretions. These studies with monoclonal antibodies demonstrated that immunologically naive animals could be protected using IgA as the sole immune agent. While the levels of protection observed in these various experimental settings were generally good, it is important to keep in mind that under natural conditions, the mucosal immune response would be polyclonal, and therefore more effective. In this respect, passive administration of colostrum rich in specific and non-specific SIgA has been shown to protect against gastrointestinal and airway infections (30, 31). In support of these numerous studies underscoring the protective function of SIgA of defined specificities, genetically modified mice unable to produce IgA, J chain, or pIgR all presented deficiencies in their capacity to fight against mucosal infectious agents (32–36).

The use of epithelial cell lines grown as polarized monolayers mimicking the mucosal barrier found in the gut and airways has proven a valuable tool to demonstrate the properties of immune exclusion exerted by IgA/SIgA toward pathogens and toxins *in vitro*. In such models, the antibody acted by blocking binding of cholera toxin (37), *C. difficile* toxin A (38), and ricin (39), thus preventing subsequent damage including fluid loss, cytotoxicity, and intoxication of exposed epithelial cells. Interference with attachment to epithelial cells and blocking of transmission of HIV from epithelial cells to peripheral blood mononuclear cells used as viral target was confirmed as a valid mechanism of action of HIV gp120-specific IgA (40). The crucial implication of the IgA isotype antibody in the process was further exemplified by the demonstration that SIgA, but not IgG, isolated from seropositive patients prevented HIV entry (41, 42). Along the same line, adhesion of enteropathogenic *Escherichia coli* strains capable of targeting epithelial cells could be inhibited by SIgA (43). When tested, polymeric IgA turned out to be systematically superior in maintaining cell integrity as compared to monomeric IgA or IgG of the specificity, indicating that the highest avidity associated with polymeric antibodies was important in the process of neutralization, possibly by favoring agglutination (38, 44).

Such an *in vitro* model has further shown its value by underscoring the ability of transcytosed SIgA to neutralize invading influenza, Sendaï, or rotaviruses intracellularly (45–48) (Figure 1, pathway 2). During their journey to the apical surface, specific polymeric IgA antibodies colocalized with viral hemagglutinin, neuraminidase, or surface viral proteins within the apical recycling endosomes, thus preventing intracellular replication or assembly, eventually resulting in reduced viral titers in the supernatant and cell lysates. Apical to basolateral transcytosis of HIV isolates across polarized epithelial cell monolayers demonstrated that HIV dissemination was blocked by polymeric IgA directed against the glycoprotein (gp)41 envelope protein, thus excluding the virus from spreading to the lamina propria (49). As for other viruses, intracellular neutralization took place inside the apical recycling endosome, and SIgA-based immune complexes were selectively recycled to the apical, lumen-like surface of the polarized monolayer. Intracellular neutralization with transcytosing IgA directed against HIV gp120, but not IgG with identical Fv domains, was accompanied by inhibition of viral replication inside epithelial cells (50). Interestingly, neutralization was dependent on the concentration of polymeric IgA added in the basolateral, serosal-like compartment, and reached a plateau which corresponded to the SIgA content in human secretions, i.e., about 100 μg/ml (51). The excretory function of SIgA appears as another plausible mechanism that contributes to microbial elimination at mucosal surfaces: when soluble polymeric IgA-based immune complexes were added to the basolateral compartment of polarized monolayers of epithelial cells expressing pIgR, the complexes were transported intact to the apical side (52). Capture of an antigen by polymeric IgA present in the lamina propria and subsequent secretion by intestinal crypt cells expressing high amount of basolateral pIgR was further demonstrated *in vivo* (53) (Figure 1, pathway 3).

From these various modes of action, one can conclude that multiple levels of SIgA-mediated protection fulfill complementary functions in order (1) to create a barrier at mucosal surfaces, (2) to eliminate within epithelial cells, or (3) to keep noxious microorganisms away from the body’s internal compartments. Although not tackled in this review, one has to keep in mind that a variety of other back-up mechanisms involving systemic IgA and FcαR1-bearing cellular partners are available to ensure efficacious protection against a myriad of pathogenic antigens (54, 55).

In human, correlation between resistance to infection and high specific SIgA titers was described in several studies dealing with immunity to *Vibrio cholera* infection. The presence of LPS-specific SIgA, as determined by antibodies measured in feces by ELISA, and in ELISPOT assays detecting antibody-secreting cells, allowed establishment of a strong association between SIgA and reduced level of infection (56, 57). Correlation between a strong mucosal IgA response and protection against influenza virus was also documented in vaccinated mice (58). Humans suffering from IgA deficiency (IgAD) exhibit increased frequency of upper respiratory and gastrointestinal tract infections (59, 60), yet the consequences are not always profound, mostly because compensatory adaptive or innate mechanisms such as the substitution of SIgM for SIgA take over (61). Moreover, assessment of the true contribution of IgA is complicated by the fact that the lack of IgA is rarely absolute and may be accompanied by deficiencies in other isotypes. The association of certain major histocompatibility complex haplotypes (62) and mutations in transmembrane activator and calcium-modulating cyclophilin ligand interactor (63) in patients with IgAD may further contribute to confusion when it comes to assigning a direct and unique role to IgA in the prevention of gastrointestinal diseases (64). In this respect, IgA-knock-out mice appear to display the same alterations in the expression of other isotypes and defects in immune responses (65, 66), thus making it difficult to draw unambiguous conclusions.

## Multiple Facets of the Functionality of SC in SIgA Antibodies

Free SC is composed of five Ig-like domains folded as compact ellipsoids stabilized internally by several disulfide bridges. Overall, the molecule displays a J-shape with all seven glycosylation sites exposed on the same surface, away from the binding site for polymeric IgA (67). The specific and stable interaction of SC with polymeric IgA in SIgA involves basically all domains, with domain 1 serving as the original anchoring site for polymeric IgA, and domains 2 and 3 spatially constraining domain 5 to ensure formation of a productive disulfide bridge with one Cα2 domain of one monomer in polymeric IgA (68, 69). Three dimensional analyses of human SIgA1 and SIgA2 subclasses shows SC wrapping domains Cα2 and Cα3 of polymeric IgA compactly, a feature that may be essential to the remarkable stability of the antibody (70). While exposure to intestinal proteases of polymeric IgA leads to rapid degradation into Fab and F(ab′)2 fragments, cleavage sites within domains Cα2 and Cα3 are masked in the presence of bound SC, resulting in a close to 24-h delay in enzymatic clipping (71, 72). Cross-protection takes place, as bound SC remains unaffected, in contrast to free SC, which is rapidly and totally degraded, to an extent similar to control IgG antibodies. Stability of SIgA is also increased upon binding of antigens of various size and nature: hierarchy shows the best protection toward proteases following interaction with a bacterium, then with a virus, and finally with a protein toxin (73). Such intrinsic properties makes SIgA well-suited to survive the hostile environment that prevails in the gut, and allowing to fulfill its protective function.

Another characteristics of bound SC in SIgA is its ability to confer hydrophilic properties to the Fc fragment of the antibody via the seven surface-exposed N-linked oligosaccharides equipped with terminal sialic acid residues. It is thought that this pattern is important for interaction with mucus, and therefore proper location of the antibody in the close proximity to the mucosal surfaces it is supposed to protect. SIgA-based immune complexes tethered within the mucus layer overlying the epithelium further limits diffusion in the luminal environment and aids their clearance from the gut via peristalsis (74). SIgA anchoring in mucus may account for the observation that the outer mucus layer is the preferential habitat for the microbiota in the colon (75). However, the identification of CD71 as a SIgA receptor on the apical surface of intestinal epithelial cells (IECs) grown in Ussing chambers (76), together with the fact that SIgA binds commensal bacteria via SC (77) suggests that a more dynamic situation occurs in this part of the gut. In a mouse model of lung infection by *Shigella flexneri*, mucus-mediated anchoring of SIgA was found to be instrumental to guarantee neutralization of the bacterium preventing entry into the tissue (78); polymeric IgA mostly found in the lumen of the nasal cavity and bronchi was inefficient at protecting the mice. Similarly, removal of carbohydrate chains of SC within SIgA molecules abolished anchoring to mucus and associated protective function (79). SC can thus be seen as an essential constituent of SIgA, in that it ensures both sustained stability and proper localization in the mucosal environment, two features instrumental to the optimal function of the antibody.

In SC, N-glycans contribute to approximately 20% of the molecular weight of the protein, and endow SIgA with further binding sites for bacterial lectin-like adhesins, in addition to the four Fab domains (80). For example, SC interacts directly with a surface protein of *Streptococcus pneumoniae*, choline binding protein A (CbpA), a bacterial factor involved in colonization of the nasopharynx of rats (81). Binding was dependent on amino acid sequences present in domains 3 and 4 of SC (82) and on a highly conserved hexapeptide motif within CbpA (83). It was proposed that such an association will serve to preclude contact with epithelial cells, yet a report using the unencapsulated *S. pneumoniae* strain Rx6 had described facilitated invasion of pIgR expressing Detroit 562 cells (84). The innate-like properties of free SC in the defense against mucosal pathogens was further demonstrated in the case of *Clostridium difficile* toxin A and enteropathogenic *Escherichia coli* intimin (85) (Figure 1, pathway 4); neutralization of the bacterial products by free SC or non-specific SIgA prevented infection of target epithelial cells via interaction with sialic and galactose residues displayed on the surface of SC. Denaturation of the free SC polypeptide scaffold brings sugar moieties in a conformation no longer able to interact with the bacterial epitopes, arguing for the possibility that finely tuned spatial disposition is important for specific recognition by complex carbohydrates.

The importance of the glycosylated nature of SC and SIgA in exerting their protective function can be further illustrated as for instance in the case of sialyloligosaccharides preventing epithelial adhesion of *Escherichia coli* through type I fimbrial lectin (86, 87). SC in SIgA recovered from human colostrum was described to inhibit adhesion of *Helicobacter pylori* to human gastric surface mucous cells in a fucose-dependent manner (88). Carbohydrate side chains in SIgA serve as a docking site for ricin toxin; human SIgA with no Fab-dependent specificity for ricin reduced attachment to the apical surface of epithelial cell lines in culture and to the luminal surfaces of human intestinal villi via SC and the IgA heavy chain (89). Although not a universal mechanism, these many examples identify free and bound SC as microbial scavengers contributing to the anti-pathogenic arsenal that protects the body epithelial surfaces.

## Interfering Effects of SIgA on Fitness of Infectious Bacteria

Blocking of interaction with epithelial cells possibly through agglutination of mucosal microorganisms may not be the only mechanism by which SIgA exerts its protective function. Recent evidence argues for a more direct effect on the bacterial viability or pathogenicity, as for example by perturbation of the bioenergetic machinery, impact on motility, disruption of virulence factors involved in bacterial entry (90). For example, in the presence of sub-agglutinating amounts of IgA specific for the O-antigen of LPS (Sal4 mAb), the capacity of *Salmonella typhimurium* to invade epithelial cell monolayers was reduced by a factor of 20 (91). In support of this observation, Fab fragments derived from the same IgA, although unable to trigger agglutination, blocked entry as efficiently as the whole antibody molecule. In addition, treatment with Sal4 led to a complete paralysis of the bacterium within 15 min, again independently of agglutination (91). These data are consistent with the idea that IgA-mediated interference with motility and entry accounts for the protective function of Sal4 in the case of *Salmonella* invasion. Further studies revealed that Sal4 treatment impaired T3SS-mediated translocon formation and attenuated the delivery of tagged effector proteins into target epithelial cells (92). Changes in surface ultrastructure, alterations in outer membrane permeability, a partial reduction in membrane energetics and intracellular ATP levels were all detected upon association of Sal4 IgA with *Salmonella*, a series of features that can render the bacterium avirulent. This occurs by triggering a cyclic dimeric guanosine monophosphate-dependent signaling pathway through YeaJ, a proposed inner membrane-localized diguanylate cyclase and a known regulator of cellulose biosynthesis. For the bacterium, this results in loss of motility due to exopolysaccharide production and biofilm formation (93). From an antibody point of view, IgA possesses the ability to convert *S. typhimurium* from an invasive, motile status to a non-motile, avirulent condition via direct impact on several metabolic pathways. A similar inhibitory mechanism occurs upon binding of a murine monoclonal IgA (IgAC5) to the O-antigen of *Shigella flexneri* serotype 5a (94): transient impairment (45–60 min) of the T3SS, which is necessary for bacterial entry into IECs is coincident with a partial reduction in the bacterial membrane potential and a decrease in intracellular ATP levels.

## The Role of SIgA in Controlling Epithelial Transport

An extension of the function of SIgA at mucosal surfaces is the importance of immune exclusion for the protection of the host against excessive antigenic challenge from environmental macromolecules. IgAD subjects with IgE-mediated atopic disease had increased allergen penetration through mucosal membranes and formation of circulating immune complexes (95, 96) initially suggested that SIgA had a role in controlling absorption of food antigens and in reducing susceptibility to atopic allergies. Experiments performed in mouse models of airway allergy supported the finding that antigen-specific SIgA suppresses features associated with inflammation and asthma (97–100). The importance of IgA in the process was further illustrated in the gut by the finding that mice sensitized with bovine lactoglobulin had much lower frequencies of IgA-producing cells in Peyer’s patches, as well as reduced fecal SIgA when compared to mice actively tolerized with the same protein (101). The production of saliva antigen-specific SIgA was consistently enhanced in a mouse model of allergic asthma in which sublingual vaccination triggered protection against subsequent challenge (102). However, allergen-specific SIgA is not always increased in successfully tolerized animals, and can even be present in large amounts in sensitized ones without conferring protection (103). Oral tolerance can be induced in pIgR knock-out mice lacking SIgA, with protection against systemic hypersensitivity ensured via compensatory Treg function (104). This series of contradictory results in allergy and inflammatory diseases adds to the continuing debate about the protective role of SIgA in these deleterious processes. Moreover, the importance of SIgA against allergic diseases remains unclear with respect to recent clinical studies. Patients with IgAD displayed increased risk of food hypersensitivity at the age of 4 years (105), whereas in another cohort, IgAD did not show any correlation with food allergy (106). Further studies are required to clarify the importance of SIgA in the maintenance of local tolerance, and eventually the integrity of the intestinal barrier.

In addition to play an essential role in immune exclusion, SIgA, in contrast to IgM and IgG, exhibits the striking ability to adhere selectively to the apical membrane of M cells overlying mouse and human Peyer’s patches (107, 108). Subsequent limited transport across the epithelium resulted in the presence of small amounts of SIgA in the M cell pocket and in processes that extend in the basal lamina (109). To date, an M cell-specific receptor ensuring controlled retro-transcytosis of SIgA has not yet been identified, although one can speculate that it needs to display particular properties (low expression, binding activity in the presence of a co-receptor, recognition of altered molecular forms of SIgA) to avoid overwhelming entry of the large excess of SIgA in the intestinal lumen. *In vivo* uptake of SIgA delivered into mouse ligated ileal loop containing a Peyer’s patch resulted in specific targeting to, and internalization by dendritic cells (DC) in the subepithelial dome region (110). *Ex vivo*, only CD11c^+^CD11b^+^ DC isolated from Peyer’s patches and draining mesenteric lymph nodes showed selective binding and internalization mimicking the *in vivo* situation (111) (Figure 1, pathway 5). Interestingly, in mucosal tissues, such DC are poor producers of IL-12 but potent inducers of IL-10 secreting T cells (112) and IgA production from naïve B cells (113). DC-SIGN was recently identified as a possible candidate for SIgA recognition by mouse DC (114), while the existence of CD89 and CD71 (transferrin receptor) has been documented on maturing human DC (115). In support of these complementary mechanisms, modulation of DC function with inhibition of IL-12 production by IgA has been recently described (116).

Such observations led to the obvious question of the immunological relevance of the transport of SIgA molecules across the M cell and its subsequent association with DC. When administered orally in the presence of the mucosal adjuvant cholera toxin (117), genetically engineered SIgA carrying a foreign epitope from *Shigella flexneri* invasin B triggered the production of both salivary and systemic antibodies specific for the bacterial antigen (118). To further assess the nature of the mucosal immune response following re-entry of SIgA across the intestinal mucosa, mice were immunized orally with heterologous SIgA consisting of mouse polymeric IgA and human SC in the absence of any adjuvant. Engineered SIgA triggered production of human SC-specific antibodies and mixed Th1/Th2 type responses, preserved or induced IL-10 and TGF-β expression in MLN, and migration and maturation of DC along the Peyer’s patch-MLN-spleen axis (119) (Figure 1, pathway 5). By comparison with human SC adjuvantized with cholera toxin, it turned out that SIgA induced low degrees of activation in a non-inflammatory context favorable to preserve local homeostasis of the gastro-intestinal tract. Neutralization of *Shigella flexneri* by SIgA led to local suppression of pro-inflammatory circuits leading to gut tissue damages, a feature resulting form the stability of the immune complex in the harsh intestinal environment (120) (Figure 1, pathway 7).

An intriguing possibility in the context of SIgA-based immune complexes would be that these latter contribute to local immunomodulation, or early in life, to educate the mucosal immune system toward a tolerogenic profile. In support of this, milk antibodies, and in particular SIgA, prevents neonatal responsiveness against commensal bacteria (121). In this respect, timely provision of a set of maternal antibodies fitting the newborn gut microbiota primarily represented by a hand-over from the mother (at least after “classical” vaginal delivery) may justify from such regulatory mechanisms. It makes a sense to speculate that maternal milk SIgA antibodies passing across the epithelium direct associated antigens to DC, and shapes the gastro-intestinal immune system both in terms of defense or tolerization during initial exposure to non-self antigenic structures. Based on the evidence of SIgA re-entry into Peyer’s patch, a broad interpretation of the data would suggest that SIgA-coated, neutralized bacteria could prime the immune system of naïve individuals within a whole population in the absence of global infection.

## The Role of SIgA in Regulating the Microbiota

More recently, SIgA has been identified as a necessary partner in maintaining the fragile balance between the triad composed of the microbiota, the IECs lining the gastro-intestinal tract and the underlying mucosal immune system. The homeostatic control taking place at gut mucosal surfaces is essential to keep billions of colonizing, and at first sight potentially harmful microorganisms in order to ensure optimal symbiosis with the host. Indeed, any potential dysfunctions can lead to the development of pathologies such as inflammatory bowel diseases (122), or affect processes of extraction of energy and digestion of otherwise unavailable sources of nutrients such as the final degradation of carbohydrates. Commensal bacteria have been directly associated with the proper development of gut-associated lymphoid tissues such as isolated lymphoid follicles (123) or with the secretion of normal levels of SIgA (124) with unknown specificity called “natural” SIgA (125). It appears that the IgA repertoire is restricted to a minimum considering the enormous varieties of antigens encountered at mucosal surfaces (126), arguing in favor of the presence of polyspecific, low affinity antibodies in intestinal secretions (127, 128). This notion was challenged by a recent study using high-throughput sequencing to investigate the shaping of the IgA repertoire (129). Analysis of more than one million V_H_ sequences revealed that the IgA repertoire comprised both highly expanded and low frequency clones which both contributed to high diversity, a phenomenon amplified with aging due to hypermutation. Similar to mice IgA sequences, human VH sequences carry numerous somatic hypermutation (130). Whether this process relies on the reutilization of germinal centers in multiple Peyer’s patches as recently identified (131) is in need of further investigation. Programed cell death protein 1 knock-out mice that have elevated numbers of Peyer’s patch Treg cells exhibit changes in the binding capacity of their SIgA, which in turn affects the nature of the commensal bacteria (132). The fact that commensal bacteria are naturally coated by SIgA in feces of humans and mice strongly suggests that this interaction is necessary to maintain a steady-state commensal colonization. Mice expressing an activation-induced cytidine deaminase hypomorph (which disrupts somatic hypermutation but still supports class switch recombination) display changes in the composition of their microbiota (133). Together, this suggests that SIgA keeps the microbiota at bay using both Fab-dependent adaptive and glycan-mediated innate immune interactions.

By using free SC and non-specific SIgA (purified from hybridoma cell lines and colostrum) serving as substitutes of natural mucosal antibodies, the molecular basis pertaining to the interaction between SIgA and intestinal resident bacteria, i.e., *Lactobacillus*, *Bifidobacteria*, *Escherichia coli*, and *Bacteroides* strains, was identified as the many glycans residues carried by SC (77). While the interaction with Gram-positive bacteria indicated the essential role of carbohydrates in the process, binding to Gram-negative bacteria was preserved whatever the molecular form of protein partner used, suggesting the involvement of different binding motifs. Poor or absent association between Gram-positive bacteria and control IgG identified the critical role of sugar moieties in SC in selective binding of the highly diverse microbiota by the whole SIgA protein.

Recognition of commensal bacteria by IECs has been recognized to play a fundamental role in mucosal homeostasis by promoting for instance cytokine release, cell expansion, and reinforcement of the barrier integrity (134–136). Further, commensal strains coated by SIgA can potentiate the responsiveness of reconstituted IEC monolayers *in vitro* (137) (Figure 1, pathway 8). Unexpectedly, association with SIgA increased the bacterial anchoring at the apical surface of IECs, resulting in the reinforcement of the barrier integrity through increased phosphorylation of tight junction proteins promoting cell-to-cell contact. In addition, secretion of pro-inflammatory cytokines/chemokines by IECs was quenched, while expression of pIgR was promoted. As pIgR is involved in transcytosis of SIgA from the basolateral to the apical pole of IECs one can conclude that commensal bacteria complexed with SIgA generate a positive feedback on pIgR expression, leading to more receptors being available for active SIgA transcytosis. This phenomenon could account for the sustained SIgA secretion resulting from commensal colonization as observed previously (138). This contributes to further defining the function of SIgA in keeping commensal bacteria at bay through a delicate balance combining appropriate neutralization and proper sensing by the IECs. Whether the presence of the transferrin receptor (CD71) capable of binding SIgA at the apical pole of IECs (76) is involved in governing binding of SIgA-commensal bacteria complexes remains to be determined. Early in life, the role of maternal SIgA may be considered of primordial importance in limiting a potential inflammation induced by primary colonization in the gut of newborns. The presence of SIgA could contribute to the initial sensing of the newly implanted microbiota and allow proper development of the immune system under non-inflammatory conditions. Such a mechanism might be relevant to the understanding of inflammatory bowel disease which is, among others, associated with deregulated inflammatory responses to intestinal bacteria (139).

While data reported above shed light on the role of SIgA in mucosal monitoring of commensals by IECs, they do not say much on how the communication with partners of the underlying immune system is established. Limited uptake of bacteria including a *Lactobacillus* and a *Bacteroides* occurs through sampling by M cells found in intestinal Peyer’s patches, and regulated entry can be promoted upon association with non-specific SIgA (140) (Figure 1, pathway 6). The almost absent transepithelial passage observed in germ-free mice having barely detectable gut SIgA can be compensated for by administration of pre-formed SIgA-bacteria complexes. Commensal bacteria given alone get coated with endogenous SIgA within 3 h, strongly suggesting that association takes place under steady-state conditions anytime, and hence participates in keeping the large majority of bacteria in the intestinal lumen. The role of SIgA in shaping the gut microbial community composition may arise from its ability to suppress expression of certain bacterial epitopes (141), and therefore favor the fitness of one species or genus over others. Selective SIgA-mediated targeting of bacteria is restricted to the tolerogenic CD11c^+^CD11b^+^CD8^−^DC subset and macrophages located in the subepithelial dome region of Peyer’s patches, indicating that the host is not ignorant of its resident commensals (140). Upon coating of commensal bacteria, natural and/or specific SIgA largely maintains luminal compartmentalization of the microbiota, while occasionally permitting rare translocation events necessary to control the continuous dialog between the host’s immune system and its resident symbionts. Commensal bacteria associated with local DC in the subepithelial dome region do not penetrate further than the draining mesenteric lymph nodes, resulting in the confinement of immune induction against the microbiota to the mucosa (142, 143). Making the systemic immune system relatively ignorant of these organisms at this stage would permit adequate stimulation in the case of sepsis. While transport of SIgA alone or in complex with protein antigens or bacteria through Peyer’s patch M cells is well established, it remains to be determined whether other transepithelial pathways including for example M cells in isolated lymphoid follicles (144), lamina propria DC snorkeling dendrites across the tight epithelium (145), Peyer’s patch DC extending dendrites around M cells (146), or Goblet cell-mediated passage (147) can account for selective sampling and targeting of cells regulating intestinal immune responses.

## Conclusion

Mucosal surfaces at the interface between the external world and the inside of the body are the primary sites of continuous challenge with potentially infectious agents, commensal bacteria, and foreign proteins. Maintenance of the integrity and selective function of these delicate epithelia implies that tightly controlled homeostasis is ensured anytime. As a consequence, depending on the nature of the stimulus, very different immunoregulatory mechanisms have to be duly activated. A prominent effector in this network, SIgA plays a crucial role in the essential communication occurring between the host’s mucosal environment and the proper sensing of harmless inhabitants or noxious pathogens/antigens (Figure 1). To fulfill this demanding multi-task function, SIgA displays several properties that extend from classical immune exclusion and permanent checking of the microbiota to local immunomodulation via intricate contacts with microorganisms, epithelial cells including enterocytes and M cells, and DC in the mucosal associated lymphoid tissue. It must be emphasized that biochemical features associated with SIgA, such as stability in an aggressive medium, anchoring in mucus, heavy glycosylation, Fab-independent recognition of antigens, transcytosis and retro-transcytosis across the intestinal epithelium all contribute to allow the antibody to perform optimally in the particular environment of mucosal surfaces.

## Conflict of Interest Statement

The authors declare that the research was conducted in the absence of any commercial or financial relationships that could be construed as a potential conflict of interest.

## References

[1] Cardinale F Friman V Carlsson B Björkander J Armenio L Hanson LA Aberrations in titre and avidity of serum IgM and IgG antibodies to microbial and food antigens in IgA deficiency. Scand J Immunol (1992) 36:279–83 10.1111/j.1365-3083.1992.tb03100.x 1502496

[2] Ferrero RL Thiberge JM Labigne A Local immunoglobulin G antibodies in the stomach may contribute to immunity against *Helicobacter* infection in mice. Gastroenterology (1997) 113:185–94 10.1016/S0016-5085(97)70094-5 9207277

[3] Giannasca PJ Zhang ZX Lei WD Boden JA Giel MA Monath TP Serum antitoxin antibodies mediate systemic and mucosal protection from *Clostridium difficile* disease in hamsters. Infect Immun (1999) 67:527–38 9916055 10.1128/iai.67.2.527-538.1999 PMC96351

[4] Neal LM McCarthy EA Morris CR Mantis NJ Vaccine-induced intestinal immunity to ricin toxin in the absence of secretory IgA. Vaccine (2011) 29:681–9 10.1016/j.vaccine.2010.11.030 21115050 PMC3034280

[5] Brandtzaeg P Prydz H Direct evidence for an integrated function of J chain and secretory component in epithelial transport of immunoglobulins. Nature (1984) 311:71–3 10.1038/311071a0 6433206

[6] Kaetzel CS The polymeric immunoglobulin receptor: bridging innate and adaptive immune responses at mucosal surfaces. Immunol Rev (2005) 206:83–99 10.1111/j.0105-2896.2005.00278.x 16048543

[7] Hughes GJ Reason AJ Savoy L Jaton J Frutiger-Hughes S Carbohydrate moieties in human secretory component. Biochim Biophys Acta (1999) 1434:86–93 10.1016/S0167-4838(99)00168-5 10556562

[8] Mestecky J Russell MW Elson CO Intestinal IgA: novel views on its function in the defence of the largest mucosal surface. Gut (1999) 44:2–5 10.1136/gut.44.1.2 9862815 PMC1760065

[9] Eibl MM Wolf HM Fürnkranz H Rosenkranz A Prevention of necrotizing enterocolitis in low-birth-weight infants by IgA-IgG feeding. N Engl J Med (1988) 319:1–7 10.1056/NEJM198807073190101 3288866

[10] Tjellström B Stenhammar L Eriksson S Magnusson KE Oral immunoglobulin A supplement in treatment of *Clostridium difficile* enteritis. Lancet (1993) 341:701–2 10.1016/0140-6736(93)90477-X 8095616

[11] Hammarström V Smith CI Hammarström L Oral immunoglobulin treatment in *Campylobacter jejuni* enteritis. Lancet (1993) 341:1036 10.1016/0140-6736(93)91136-A 8096933

[12] Giraudi V Riganti C Torales MR Sédola H Gaddi E Upper respiratory infections in children: response to endonasal administration of IgA. Int J Pediatr Otorhinolaryngol (1997) 39:103–10 10.1016/S0165-5876(96)01472-3 9104618

[13] Heikkinen T Ruohola A Ruuskanen O Waris M Uhari M Hammarström L Intranasally administered immunoglobulin for the prevention of rhinitis in children. Pediatr Infect Dis J (1998) 17:367–72 10.1097/00006454-199805000-00004 9613647

[14] Ma JK Hikmat BY Wycoff K Vine ND Chargelegue D Yu L Characterization of a recombinant plant monoclonal secretory antibody and preventive immunotherapy in humans. Nat Med (1998) 4:601–6 10.1038/nm0598-601 9585235

[15] Weltzin R Monath TP Intranasal antibody prophylaxis for protection against viral disease. Clin Microbiol Rev (1999) 12:383–93 10398671 10.1128/cmr.12.3.383 PMC100244

[16] Zeitlin L Cone RA Whaley KJ Using monoclonal antibodies to prevent mucosal transmission of epidemic infectious diseases. Emerg Infect Dis (1999) 5:54–64 10.3201/eid0501.990107 10081672 PMC2627706

[17] Corthésy B Recombinant secretory immunoglobulin A in passive immunotherapy: linking immunology and biotechnology. Curr Pharm Biotechnol (2003) 4:51–67 10.2174/1389201033378020 12570682

[18] Michetti P Mahan MJ Slauch JM Mekalanos JJ Neutra MR Monoclonal secretory immunoglobulin A protects mice against oral challenge with the invasive pathogen *Salmonella typhimurium*. Infect Immun (1992) 60:1786–92 1373399 10.1128/iai.60.5.1786-1792.1992 PMC257074

[19] Iankov ID Petrov DP Mladenov IV Haralambieva IH Kalev OK Balabanova MS Protective efficacy of IgA monoclonal antibodies to O and H antigens in a mouse model of intranasal challenge with *Salmonella enterica* serotype enteritidis. Microbes Infect (2004) 6:901–10 10.1016/j.micinf.2004.05.007 15310466

[20] Apter FM Michetti P Winner LS III Mack JA Mekalanos JJ Neutra MR Analysis of the roles of antilipopolysaccharide and anti-cholera toxin immunoglobulin A (IgA) antibodies in protection against *Vibrio cholerae* and cholera toxin by use of monoclonal IgA antibodies in vivo. Infect Immun (1993) 61:5279–85 8225601 10.1128/iai.61.12.5279-5285.1993 PMC281312

[21] Phalipon A Kaufmann M Michetti P Cavaillon JM Huerre M Sansonetti P Monoclonal immunoglobulin A antibody directed against serotype-specific epitope of *Shigella flexneri* lipopolysaccharide protects against murine experimental shigellosis. J Exp Med (1995) 182:769–78 10.1084/jem.182.3.769 7544397 PMC2192169

[22] Blanchard TG Czinn SJ Maurer R Thomas WD Soman G Nedrud JG Urease-specific monoclonal antibodies prevent *Helicobacter felis* infection in mice. Infect Immun (1995) 63:1394–9 7890401 10.1128/iai.63.4.1394-1399.1995 PMC173165

[23] Weltzin R Traina-Dorge V Soike K Zhang JY Mack P Soman G Intranasal monoclonal IgA antibody to respiratory syncytial virus protects rhesus monkeys against upper and lower respiratory tract infection. J Infect Dis (1996) 174:256–61 10.1093/infdis/174.2.256 8699052

[24] Renegar KB Small PA Passive transfer of local immunity to influenza-virus infection by IgA antibody. J Immunol (1991) 146:1972–8 2005388

[25] Silvey KJ Hutchings AB Vajdy M Petzke MM Neutra MR Role of immunoglobulin A in protection against reovirus entry into Murine Peyer’s patches. J Virol (2001) 75:10870–9 10.1128/JVI.75.22.10870-10879.2001 11602727 PMC114667

[26] Hutchings AB Helander A Silvey KJ Chandran K Lucas WT Nibert ML Secretory immunoglobulin A antibodies against the sigma1 outer capsid protein of reovirus type 1 Lang prevent infection of mouse Peyer’s patches. J Virol (2004) 78:947–57 10.1128/JVI.78.2.947-957.2004 14694126 PMC368743

[27] Renegar KB Small PA Jr Boykins LG Wright PF Role of IgA versus IgG in the control of influenza viral infection in the murine respiratory tract. J Immunol (2004) 173:1978–86 15265932 10.4049/jimmunol.173.3.1978

[28] Winner L III Mack J Weltzin R Mekalanos JJ Kraehenbuhl JP Neutra MR New model for analysis of mucosal immunity: intestinal secretion of specific monoclonal immunoglobulin A from hybridoma tumors protects against *Vibrio cholerae* infection. Infect Immun (1991) 59:977–82 1705246 10.1128/iai.59.3.977-982.1991 PMC258355

[29] Burns JW Siadat-Pajouh M Krishnaney AA Greenberg HB Protective effect of rotavirus VP6-specific IgA monoclonal antibodies that lack neutralizing activity. Science (1996) 272:104–7 10.1126/science.272.5258.104 8600516

[30] Wijburg OL Uren TK Simpfendorfer K Johansen FE Brandtzaeg P Strugnell RA Innate secretory antibodies protect against natural *Salmonella typhimurium* infection. J Exp Med (2006) 203:21–6 10.1084/jem.20052093 16390940 PMC2118088

[31] Alvarez N Otero O Camacho F Borrero R Tirado Y Puig A Passive administration of purified secretory IgA from human colostrum induces protection against *Mycobacterium tuberculosis* in a murine model of progressive pulmonary infection. BMC Immunol (2013) 14:S3 10.1186/1471-2172-14-S1-S3 23458564 PMC3582447

[32] Lycke N Erlandsson L Ekman L Schön K Leanderson T Lack of J chain inhibits the transport of gut IgA and abrogates the development of intestinal antitoxic protection. J Immunol (1999) 163:913–9 10395687

[33] Schwartz-Cornil I Benureau Y Greenberg H Hendrickson BA Cohen J Heterologous protection induced by the inner capsid proteins of rotavirus requires transcytosis of mucosal immunoglobulins. J Virol (2002) 76:8110–7 10.1128/JVI.76.16.8110-8117.2002 12134016 PMC155125

[34] Sun K Johansen FE Eckmann L Metzger DW An important role for polymeric Ig receptor-mediated transport of IgA in protection against *Streptococcus pneumoniae* nasopharyngeal carriage. J Immunol (2004) 173:4576–81 15383591 10.4049/jimmunol.173.7.4576

[35] Davids BJ Palm JE Housley MP Smith JR Andersen YS Martin MG Polymeric immunoglobulin receptor in intestinal immune defense against the lumen-dwelling protozoan parasite *Giardia*. J Immunol (2006) 177:6281–90 17056558 10.4049/jimmunol.177.9.6281

[36] Blutt SE Miller AD Salmon SL Metzger DW Conner ME IgA is important for clearance and critical for protection from rotavirus infection. Mucosal Immunol (2012) 5:712–9 10.1038/mi.2012.51 22739233 PMC3461240

[37] Apter FM Lencer WI Finkelstein RA Mekalanos JJ Neutra MR Monoclonal immunoglobulin A antibodies directed against cholera toxin prevent the toxin-induced chloride secretory response and block toxin binding to intestinal epithelial cells in vitro. Infect Immun (1993) 61:5271–8 7693598 10.1128/iai.61.12.5271-5278.1993 PMC281311

[38] Stubbe H Berdoz J Kraehenbuhl JP Corthésy B Polymeric IgA is superior to monomeric IgA and IgG carrying the same variable domain in preventing *Clostridium difficile* toxin A damaging of T84 monolayers. J Immunol (2000) 164:1952–60 10657645 10.4049/jimmunol.164.4.1952

[39] Mantis NJ McGuinness CR Sonuyi O Edwards G Farrant SA Immunoglobulin A antibodies against ricin A and B subunits protect epithelial cells from ricin intoxication. Infect Immun (2006) 74:3455–62 10.1128/IAI.02088-05 16714576 PMC1479255

[40] Mantis NJ Palaia J Hessell AJ Mehta S Zhu Z Corthésy B Inhibition of HIV-1 infectivity and epithelial cell transfer by human monoclonal IgG and IgA antibodies carrying the b12 V region. J Immunol (2007) 179:3144–52 17709529 10.4049/jimmunol.179.5.3144 PMC2881690

[41] Devito C Broliden K Kaul R Svensson L Johansen K Kiama P Mucosal and plasma IgA from HIV-1-exposed uninfected individuals inhibit HIV-1 transcytosis across human epithelial cells. J Immunol (2000) 165:5170–6 11046049 10.4049/jimmunol.165.9.5170

[42] Alfsen A Iniguez P Bouguyon E Bomsel M Secretory IgA specific for a conserved epitope on gp41 envelope glycoprotein inhibits epithelial transcytosis of HIV-1. J Immunol (2001) 166:6257–65 11342649 10.4049/jimmunol.166.10.6257

[43] Cravioto A Tello A Villafán H Ruiz J del Vedovo S Neeser JR Inhibition of localized adhesion of enteropathogenic *Escherichia coli* to HEp-2 cells by immunoglobulin and oligosaccharide fractions of human colostrum and breast milk. J Infect Dis (1991) 163:1247–55 10.1093/infdis/163.6.1247 1903799

[44] Renegar KB Jackson GD Mestecky J In vitro comparison of the biologic activities of monoclonal monomeric IgA, polymeric IgA, and secretory IgA. J Immunol (1998) 160:1219–23 9570537

[45] Mazanec MB Coudret CL Fletcher DR Intracellular neutralization of influenza virus by immunoglobulin A anti-hemagglutinin monoclonal antibodies. J Virol (1995) 69:1339–43 7815518 10.1128/jvi.69.2.1339-1343.1995 PMC188717

[46] Fujioka H Emancipator SN Aikawa M Huang DS Blatnik F Karban T Immunocytochemical colocalization of specific immunoglobulin A with sendai virus protein in infected polarized epithelium. J Exp Med (1998) 188:1223–9 10.1084/jem.188.7.1223 9763601 PMC2212485

[47] Ruggeri FM Johansen K Basile G Kraehenbuhl JP Svensson L Antirotavirus immunoglobulin A neutralizes virus in vitro after transcytosis through epithelial cells and protects infant mice from diarrhea. J Virol (1998) 72:2708–14 9525588 10.1128/jvi.72.4.2708-2714.1998 PMC109713

[48] Corthésy B Benureau Y Perrier C Fourgeux C Parez N Greenberg H Rotavirus anti-VP6 secretory immunoglobulin A contributes to protection via intracellular neutralization but not via immune exclusion. J Virol (2006) 80:10692–9 10.1128/JVI.00927-06 16956954 PMC1641769

[49] Bomsel M Heyman M Hocini H Lagaye S Belec L Dupont C Intracellular neutralization of HIV transcytosis across tight epithelial barriers by anti-HIV envelope protein dIgA or IgM. Immunity (1998) 9:277–87 10.1016/S1074-7613(00)80610-X 9729048

[50] Huang YT Wright A Gao X Kulick L Yan H Lamm ME Intraepithelial cell neutralization of HIV-1 replication by IgA. J Immunol (2005) 174:4828–35 15814709 10.4049/jimmunol.174.8.4828

[51] Jackson S Mestecky J Moldoveanu Z Spearman P Collection and processing of human mucosal secretions. In: Ogra PL Mestecky J Lamm ME Strober W Bienenstock J McGhee JR editors. Mucosal Immunology. San Diego, FL: Academic Press (1999). p. 1567–75

[52] Kaetzel CS Robinson JK Chintalacharuvu KR Vaerman JP Lamm ME The polymeric immunoglobulin receptor (secretory component) mediates transport of immune complexes across epithelial cells: a local defense function for IgA. Proc Natl Acad Sci U S A (1991) 88:8796–800 10.1073/pnas.88.19.8796 1924341 PMC52597

[53] Robinson JK Blanchard TG Levine AD Emancipator SN Lamm ME A mucosal IgA-mediated excretory immune system in vivo. J Immunol (2001) 166:3688–92 11238608 10.4049/jimmunol.166.6.3688

[54] Monteiro RC van de Winkel JG IgA Fc receptors. Annu Rev Immunol (2003) 21:177–204 10.1146/annurev.immunol.21.120601.141011 12524384

[55] Bakema JE van Egmond M The human immunoglobulin A Fc receptor FcαRI: a multifaceted regulator of mucosal immunity. Mucosal Immunol (2011) 4:612–24 10.1038/mi.2011.36 21937986

[56] Uddin T Harris JB Bhuiyan TR Shirin T Uddin MI Khan AI Mucosal immunologic responses in cholera patients in Bangladesh. Clin Vaccine Immunol (2011) 18:506–12 10.1128/CVI.00481-10 21248157 PMC3067383

[57] Johnson RA Uddin T Aktar A Mohasin M Alam MM Chowdhury F Comparison of immune responses to the O-specific polysaccharide and lipopolysaccharide of *Vibrio cholerae* O1 in Bangladeshi adult patients with cholera. Clin Vaccine Immunol (2012) 19:1712–21 10.1128/CVI.00321-12 22993410 PMC3491541

[58] Eliasson DG Helgeby A Schön K Nygren C El-Bakkouri K Fiers W A novel non-toxic combined CTA1-DD and ISCOMS adjuvant vector for effective mucosal immunization against influenza virus. Vaccine (2011) 29:3951–61 10.1016/j.vaccine.2011.03.090 21481325

[59] Shkalim V Monselize Y Segal N Zan-Bar I Hoffer V Garty BZ Selective IgA deficiency in children in Israel. J Clin Immunol (2010) 30:761–5 10.1007/s10875-010-9438-x 20571893

[60] Priyadarsi A Sankar J H1N1 infection associated with persistent lower respiratory tract illness in an infant with isolated IgA deficiency. BMJ Case Rep (2012). 10.1136/bcr.11.2011.5132 22665468 PMC3291016

[61] Nilssen DE Friman V Theman K Björkander J Kilander A Holmgren J B-cell activation in duodenal mucosa after oral cholera vaccination in IgA-deficient subjects with or without IgG subclass deficiency. Scand J Immunol (1993) 38:201–8 10.1111/j.1365-3083.1993.tb01714.x 8346420

[62] Wang N Hammarström L IgA deficiency: what is new? Curr Opin Clin Immunol (2012) 12:602–8 10.1097/ACI.0b013e3283594219 23026772

[63] Martinez-Gallo M Radigan L Almejún MB Martínez-Pomar N Matamoros N Cunningham-Rundles C TACI mutations and impaired B-cell function in subjects with CVID and in healthy heterozygotes. J Allergy Clin Immunol (2013) 131:468–76 10.1016/j.jaci.2012.10.029 23237420 PMC3646641

[64] Agarwal S Mayer L Pathogenesis and treatment of gastrointestinal disease in antibody deficiency syndrome. J Allergy Clin Immunol (2009) 142:658–64 10.1016/j.jaci.2009.06.018 19665769 PMC3882760

[65] Arulanandam BP Raeder RH Nedrud JG Bucher DJ Le J Metzger DW IgA immunodeficiency leads to inadequate Th cell priming and increased susceptibility to influenza virus infection. J Immunol (2001) 166:226–31 11123296 10.4049/jimmunol.166.1.226

[66] Duchez S Amin R Cogné N, Delpy L Sirac C Pascal V Premature replacement of mu with alpha immunoglobulin chains impairs lymphopoiesis and mucosal homing but promotes plasma cell maturation. Proc Natl Acad Sci U S A (2010) 107:3064–9 10.1073/pnas.0912393107 20133609 PMC2840347

[67] Bonner A Perrier C Corthésy B Perkins SJ Solution structure of human secretory component and implications for biological function. J Biol Chem (2007) 282:16969–80 10.1074/jbc.M701281200 17428798

[68] Bakos MA Kurosky A Goldblum RM Characterization of a critical binding site for human polymeric Ig on secretory component. J Immunol (1991) 147:3419–26 1940346

[69] Crottet P Corthésy B Mapping the interaction between murine IgA and murine secretory component carrying epitope substitutions reveals a role of domains II and III in covalent binding to IgA. J Biol Chem (1999) 274:31456–62 10.1074/jbc.274.44.31456 10531347

[70] Bonner A Almogren A Furtado PB Kerr MA Perkins SJ The nonplanar secretory IgA2 and near planar secretory IgA1 solution structures rationalize their different mucosal immune responses. J Biol Chem (2009) 284:5077–87 10.1074/jbc.M807529200 19109255 PMC2643523

[71] Crottet P Corthésy B Secretory component delays the conversion of secretory IgA into antigen-binding competent F(ab′)2: a possible implication for mucosal defense. J Immunol (1998) 161:5445–53 9820520

[72] Longet S Miled S Lötscher M Miescher SM Zuercher AW Corthésy B Human plasma-derived polymeric IgA and IgM antibodies associate with recombinant secretory component to yield biologically active secretory-like antibodies. J Biol Chem (2013) 288:4085–94 10.1074/jbc.M112.410811 23250751 PMC3567660

[73] Duc M Johansen FE Corthésy B Antigen binding to secretory immunoglobulin A results in decreased sensitivity to intestinal proteases and increased binding to cellular Fc receptors. J Biol Chem (2010) 285:953–60 10.1074/jbc.M109.059220 19910466 PMC2801296

[74] Deplancke B Gaskins HR Microbial modulation of innate defense: goblet cells and the intestinal mucus layer. Am J Clin Nutr (2001) 73:1131S–41 11393191 10.1093/ajcn/73.6.1131S

[75] Johansson ME Larsson JM Hansson GC The two mucus layers of colon are organized by the MUC2 mucin, whereas the outer layer is a legislator of host-microbial interactions. Proc Natl Acad Sci U S A (2011) 108:4659–65 10.1073/pnas.1006451107 20615996 PMC3063600

[76] Lebreton C Ménard S Abed J Moura IC Coppo R Dugave C Interactions among secretory immunoglobulin A, CD71, and transglutaminase-2 affect permeability of intestinal epithelial cells to gliadin peptides. Gastroenterology (2012) 143:698–707 10.1053/j.gastro.2012.05.051 22750506

[77] Mathias A Corthésy B Recognition of gram-positive intestinal bacteria by hybridoma- and colostrum-derived secretory immunoglobulin A is mediated by carbohydrates. J Biol Chem (2011) 286:17239–47 10.1074/jbc.M110.209015 21454510 PMC3089566

[78] Phalipon A Cardona A Kraehenbuhl JP Edelman L Sansonetti PJ Corthésy B Secretory component: a new role in secretory IgA-mediated immune exclusion in vivo. Immunity (2002) 17:107–15 10.1016/S1074-7613(02)00341-2 12150896

[79] Murthy AK Chagenty BK Troutman T Guentzel MN Yu JJ Ali SK Mannose-containing oligosaccharides of non-specific human secretory immunoglobulin A mediate inhibition of Vibrio cholerae biofilm formation. PLoS One (2011) 6:e16847 10.1371/journal.pone.0016847 21347387 PMC3036728

[80] Royle L Roos A Harvey DJ Wormald MR van Gijlswijk-Janssen D el-Redwan RM Secretory IgA N- and O-glycans provide a link between the innate and adaptive immune systems. J Biol Chem (2003) 278:20140–53 10.1074/jbc.M301436200 12637583

[81] Hammerschmidt S Talay SR Brandtzaeg P Chhatwal GS SpsA, a novel pneumococcal surface protein with specific binding to secretory immunoglobulin A and secretory component. Mol Microbiol (1997) 25:1113–24 10.1046/j.1365-2958.1997.5391899.x 9350867

[82] Lu L Lamm ME Li H Corthésy B Zhang JR The human polymeric immunoglobulin receptor binds to *Streptococcus pneumoniae* via domains 3 and 4. J Biol Chem (2003) 278:48178–87 10.1074/jbc.M306906200 13679368

[83] Luo R Mann B Lewis WS Rowe A Heath R Stewart ML Solution structure of choline binding protein A, the major adhesin of streptococcus pneumoniae. EMBO J (2005) 24:34–43 15616594 10.1038/sj.emboj.7600490 PMC544903

[84] Zhang JR Mostov KE Lamm ME Nanno M Shimida S Ohwaki M The polymeric immunoglobulin receptor translocates pneumococci across human nasopharyngeal epithelial cells. Cell (2000) 102:827–37 10.1016/S0092-8674(00)00071-4 11030626

[85] Perrier C Sprenger N Corthésy B Glycans on secretory component participate in innate protection against mucosal pathogens. J Biol Chem (2006) 281:14280–7 10.1074/jbc.M512958200 16543244

[86] Wold AE Mestecky J Tomana M Kobata A Ohbayashi H Endo T Secretory immunoglobulin A carries oligosaccharide receptors for *Escherichia coli* type 1 fimbrial lectin. Infect Immun (1990) 58:3073–7 2201644 10.1128/iai.58.9.3073-3077.1990 PMC313613

[87] Schroten H Stapper C Plogmann R Köhler H Hackre J Hanisch FG Fab-independent antiadhesion effects of secretory immunoglobulin A on S-fimbriated *Escherichia coli* are mediated by sialyloligosaccharides. Infect Immun (1998) 66:3971–3 9673289 10.1128/iai.66.8.3971-3973.1998 PMC108467

[88] Borén T Falk P Roth KA Larson G Normark S Attachment of *Helicobacter pylori* to human gastric epithelium mediated by blood group antigens. Science (1993) 262:1892–5 10.1126/science.8018146 8018146

[89] Mantis NJ Farrant SA Mehta S Oligosaccharide side chains on human secretory IgA serve as receptors for ricin. J Immunol (2004) 172:6838–45 15153502 10.4049/jimmunol.172.11.6838

[90] Mantis NJ Forbes SJ Secretory IgA: arresting microbial pathogens at epithelial borders. Immunol Invest (2010) 39:383–406 10.3109/08820131003622635 20450284 PMC3774547

[91] Forbes SJ Eschmann M Mantis NJ Inhibition of *Salmonella enterica* serovar typhimurium motility and entry into epithelial cells by a protective antilipopolysaccharide monoclonal immunoglobulin A antibody. Infect Immun (2008) 76:4137–44 10.1128/IAI.00416-08 18625740 PMC2519396

[92] Forbes SJ Martinelli D Hsieh C Ault JG Marko M Mannella CA Association of a protective monoclonal IgA with the O antigen of *Salmonella enterica* serovar Typhimurium impacts type 3 secretion and outer membrane integrity. Infect Immun (2012) 80:2454–63 10.1128/IAI.00018-12 22473607 PMC3416483

[93] Amarasinghe JJ D’Hondt RE Waters CM Mantis NJ Exposure of *Salmonella enterica* *Serovar typhimurium* to a protective monoclonal IgA triggers exopolysaccharide production via a diguanylate cyclase-dependent pathway. Infect Immun (2013) 81:653–64 10.1128/IAI.00813-12 23230292 PMC3584880

[94] Forbes SJ Bumpus T McCarthy EA Corthésy B Mantis NJ Transient suppression of *Shigella flexneri* type 3 secretion by a protective O-antigen-specific monoclonal IgA. MBio (2011) 2:e00042–11 10.1128/mBio.00042-11 21610121 PMC3101778

[95] Stokes CR Taylor B Turner MW Association of house-dust and grass-pollen allergies with specific IgA antibody deficiency. Lancet (1974) 2:485–8 10.1016/S0140-6736(74)92014-5 4136547

[96] Cunningham-Rundles C Brandeis WE Good RA Day NK Milk precipitins, circulating immune complexes and IgA deficiency. Adv Exp Med Biol (1978) 107:523–30 10.1007/978-1-4684-3369-2_59 570344

[97] Schwarze J Cieslewicz G Joetham A Sun LK Sun WN Chang TW Antigen-specific immunoglobulin A prevents increased airway responsiveness and lung eosinophilia after airway challenge in sensitized mice. Am J Respir Crit Care Med (1998) 158:519–25 10.1164/ajrccm.158.2.9801014 9700130

[98] Pilette C Nouri-Aria KT Jacobson MR Wolcock LK Detry B Walker SM Grass pollen immunotherapy induces an allergen-specific IgA2 antibody response associated with mucosal TGF-β expression. J Immunol (2007) 178:4658–66 17372025 10.4049/jimmunol.178.7.4658

[99] Hajek AR Lindley AR Favoreto S Carter R Schleimer RP Kuperman DA 12/15 lipoxygenase deficiency protects mice from allergic airways inflammation and increases secretory IgA levels. J Allergy Clin Immunol (2008) 122:633–9 10.1016/j.jaci.2008.06.021 18692885 PMC2802267

[100] Smits HH Gloudemans AK van Nimwegen M Willart MA Soullié T, Muskens F Cholera toxin B suppresses allergic inflammation through induction of secretory IgA. Mucosal Immunol (2009) 2:331–9 10.1038/mi.2009.16 19404246

[101] Frossard CP Hauser C Eigenmann PA Antigen-specific secretory IgA antibodies in the gut are decreased in a mouse model of food allergy. J Allergy Clin Immunol (2004) 114:377–82 10.1016/j.jaci.2004.03.040 15316519

[102] Tourdot S Airouche S Berjont N Moussu H Betbeder D Nony E Efficacy of sublingual vectorized recombinant Bet v 1a in a mouse model of birch pollen allergic asthma. Vaccine (2013) 31:2628–37 pii: S0264-410X(13)00378-2, 10.1016/j.vaccine.2013.03.041 23583462

[103] Perrier C Thierry A-C Mercenier A Corthésy B Allergen-specific antibody and cytokine responses, mast cell reactivity and intestinal permeability upon oral challenge of sensitized and tolerized mice. Clin Exp Allergy (2009) 40:153–62 10.1111/j.1365-2222.2009.03329.x 19689461

[104] Karlsson MR Johansen FE Kahu H Macpherson AJ Brandtzaeg P Hypersensitivity and oral tolerance in the absence of a secretory immune system. Allergy (2010) 65:561–70 10.1111/j.1398-9995.2009.02225.x 19886928

[105] Janzi M Kull I Sjöberg R Wan J Melén E Bayat N Selective IgA deficiency in early life: association to infections and allergic diseases during childhood. Clin Immunol (2009) 133:78–85 10.1016/j.clim.2009.05.014 19541543

[106] Aghamohammadi A Cheraghi T Gharagozlou M Movahedi M Rezaei N Yeganeh M IgA deficiency: correlation between clinical and immunological phenotypes. J Clin Immunol (2009) 29:130–6 10.1007/s10875-008-9229-9 18683032

[107] Roy MJ Varvayanis M Development of dome epithelium in gut-associated lymphoid tissues: association of IgA with M cells. Cell Tissue Res (1987) 248:645–51 10.1007/BF00216495 3300998

[108] Mantis NJ Cheung MC Chintalacharuvu KR Rey J Corthésy B Neutra MR Selective adherence of IgA to murine Peyer’s patch M cells: evidence for a novel IgA receptor. J Immunol (2002) 169:1844–51 12165508 10.4049/jimmunol.169.4.1844

[109] Weltzin R Lucia-Jandris P Michetti P Fields BN Kraehenbuhl JP Neutra MR Binding and transepithelial transport of immunoglobulins by intestinal M cells: demonstration using monoclonal IgA antibodies against enteric viral proteins. J Cell Biol (1989) 108:1673–85 10.1083/jcb.108.5.1673 2541137 PMC2115566

[110] Rey J Garin N Spertini F Corthésy B Targeting of secretory IgA to Peyer’s patch dendritic and T cells after transport by intestinal M cells. J Immunol (2004) 172:3026–33 14978107 10.4049/jimmunol.172.5.3026

[111] Kadaoui K Corthésy B Secretory IgA mediates bacterial translocation to dendritic cells in mouse Peyer’s patches with restriction to mucosal compartment. J Immunol (2007) 179:7751–7 18025221 10.4049/jimmunol.179.11.7751

[112] Chirdo FG Millington OR Beacock-Sharp H Mowat AM Immunomodulatory dendritic cells in intestinal lamina propria. Eur J Immunol (2005) 35:1831–40 10.1002/eji.200425882 16010704

[113] Sato A Hashiguchi M Toda E Iwasaki A Hachimura S Kaminogawa S CD11b^+^ Peyer’s patch dendritic cells secrete IL-6 and induce IgA secretion from naive B cells. J Immunol (2003) 171:3684–90 14500666 10.4049/jimmunol.171.7.3684

[114] Baumann J Park CG Mantis NJ Recognition of secretory IgA by DC-SIGN: implications for immune surveillance in the intestine. Immunol Lett (2010) 131:59–66 10.1016/j.imlet.2010.03.005 20362001 PMC2954462

[115] Pasquier B Lepelletier Y Baude C Hermine O Monteiro RC Differential expression and function of IgA receptors (CD89 and CD71) during maturation of dendritic cells. J Leukoc Biol (2004) 76:1134–41 10.1189/jlb.0204101 15371488

[116] Lecocq M Detry B Guisset A Pilette C FcαRI-mediated inhibition of IL-12 production and priming by IFN-γ of human monocytes and dendritic cells. J Immunol (2013) 190:2362–71 10.4049/jimmunol.1201128 23359507

[117] Lycke N From toxin to adjuvant: basic mechanisms for the control of mucosal IgA immunity and tolerance. Immunol Lett (2005) 97:193–8 10.1016/j.imlet.2004.12.008 15752558

[118] Corthésy B Kaufmann M Phalipon A Peitsch M Neutra MR Kraehenbuhl JP A pathogen-specific epitope inserted into recombinant secretory immunoglobulin A is immunogenic by the oral route. J Biol Chem (1996) 271:33670–7 8969237 10.1074/jbc.271.52.33670

[119] Favre LI Spertini F Corthésy B Secretory IgA possesses intrinsic modulatory properties stimulating mucosal and systemic immune responses. J Immunol (2005) 175:2793–800 16116164 10.4049/jimmunol.175.5.2793

[120] Boullier S Tanguy M Kadaoui KA Caubet C Sansonetti P Corthésy B Secretory IgA-mediated neutralization of *Shigella flexneri* prevents intestinal tissue destruction by down-regulating inflammatory circuits. J Immunol (2009) 183:5879–85 10.4049/jimmunol.0901838 19828639

[121] Harris NL Spoerri I Schopfer JF Nembrini C Merky P Massacand J Mechanisms of neonatal mucosal antibody protection. J Immunol (2006) 177:6256–62 17056555 10.4049/jimmunol.177.9.6256

[122] Nell S Suerbaum S Josenhans C The impact of the microbiota on the pathogenesis of IBD: lessons from mouse infection models. Nat Rev Microbiol (2010) 8:564–77 10.1038/nrmicro2403 20622892

[123] Eberl G Lochner M The development of intestinal lymphoid tissues at the interface of self and microbiota. Mucosal Immunol (2009) 2:478–85 10.1038/mi.2009.114 19741595

[124] Suzuki K Meek B Doi Y Muramatsu M Chiba T Honjo T Aberrant expansion of segmented filamentous bacteria in IgA-deficient gut. Proc Natl Acad Sci U S A (2004) 101:1981–6 10.1073/pnas.0307317101 14766966 PMC357038

[125] Quan CP Berneman A Pires R Avrameas S Bouvet JP Natural polyreactive secretory immunoglobulin A autoantibodies as a possible barrier to infection in humans. Infect Immun (1997) 65:3997–4004 9316998 10.1128/iai.65.10.3997-4004.1997 PMC175574

[126] Stoel M Jiang HQ van Diemen CC Bun JC Dammers PM Thurnheer MC Restricted IgA repertoire in both B-1 and B-2 cell-derived gut plasmablasts. J Immunol (2005) 174:1046–54 15634929 10.4049/jimmunol.174.2.1046

[127] Bos NA Jiang HQ Cebra JJ T cell control of the gut IgA response against commensal bacteria. Gut (2001) 48:762–4 10.1136/gut.48.6.762 11358892 PMC1728317

[128] Slack E Balmer ML Fritz JH Hapfelmeier S Functional flexibility of intestinal IgA – broadening the fine line. Front Immunol (2012) 3:100 10.3389/fimmu.2012.00100 22563329 PMC3342566

[129] Lindner C Wahl B Föhse L Suerbaum S Macpherson AJ Prinz I Age, microbiota, and T cells shape diverse individual IgA repertoires in the intestine. J Exp Med (2012) 209:365–77 10.1084/jem.20111980 22249449 PMC3280880

[130] Barone F Vossenkamper A Boursier L Su W Watson A John S IgA-producing plasma cells originate from germinal centers that are induced by B-cell receptor engagement in humans. Gastroenterology (2011) 140:947–56 10.1053/j.gastro.2010.12.005 21147106 PMC7115992

[131] Bergqvist P Stensson A Hazanov L Holmberg A Mattsson J Mehr R Re-utilization of germinal centers in multiple Peyer’s patches results in highly synchronized, oligoclonal, and affinity-matured gut IgA responses. Mucosal Immunol (2013) 6:122–35 10.1038/mi.2012.56 22785230

[132] Kawamoto S Tran TH Maruya M Suzuki K Doi Y Tsutsuim Y The inhibitory receptor PD-1 regulates IgA selection and bacterial composition in the gut. Science (2012) 336:485–9 10.1126/science.1217718 22539724

[133] Wei M Shinkura R Doy Y Mayura M Fagarasan S Honjo T Mice carrying a knock-in mutation of Aicda resulting in a defect in somatic hypermutation have impaired gut homeostasis and compromised mucosal defense. Nat Immunol (2011) 12:264–70 10.1038/ni.1991 21258321

[134] Negishi H Miki S Sarashina H Taguchi-Atarashi N Nakajima A Matsuki K Essential contribution of IRF3 to intestinal homeostasis and microbiota-mediated Tslp gene induction. Proc Natl Acad Sci U S A (2012) 109:21016–21 10.1073/pnas.1219482110 23213237 PMC3529020

[135] Nishio J Honda K Immunoregulation by the gut microbiota. Cell Mol Life Sci (2012) 69:3635–50 10.1007/s00018-012-0993-6 22527722 PMC11114866

[136] Yu LC Wang JT Wei SC Ni YH Host-microbial interactions and regulation of intestinal epithelial barrier function: from physiology to pathology. World J Gastrointest Pathophysiol (2012) 3:27–43 10.4291/wjgp.v3.i1.27 22368784 PMC3284523

[137] Mathias A Duc M Favre L Benyacoub J Blum S Corthésy B Potentiation of polarized intestinal Caco-2 cell responsiveness to probiotics complexed with secretory IgA. J Biol Chem (2010) 285:33906–13 10.1074/jbc.M110.135111 20729211 PMC2962490

[138] Shroff KE Meslin K Cebra JJ Commensal enteric bacteria engender a self-limiting humoral mucosal immune response while permanently colonizing the gut. Infect Immun (1995) 63:3904–13 7558298 10.1128/iai.63.10.3904-3913.1995 PMC173549

[139] Swidsinski A Weber J Loening-Baucke V Hale LP Lochs H Spatial organization and composition of the mucosal flora in patients with inflammatory bowel disease. J Clin Microbiol (2005) 43:3380–9 10.1128/JCM.43.7.3380-3389.2005 16000463 PMC1169142

[140] Rol N Favre L Benyacoub J Corthésy B The role of secretory immunoglobulin A in the natural sensing of commensal bacteria by mouse Peyer’s patch dendritic cells. J Biol Chem (2012) 287:40074–82 10.1074/jbc.M112.405001 23027876 PMC3501041

[141] Peterson DA McNulty NP Guruge JL Gordon JI IgA response to symbiotic bacteria as a mediator of gut homeostasis. Cell Host Microbe (2007) 2:328–39 10.1016/j.chom.2007.09.013 18005754

[142] Macpherson AJ Uhr T Induction of protective IgA by intestinal dendritic cells carrying commensal bacteria. Science (2004) 303:1662–5 10.1126/science.1091334 15016999

[143] Macpherson AJ Slack E Geuking MB McCoy KD The mucosal firewalls against commensal intestinal microbes. Semin Immunopathol (2009) 31:145–9 10.1007/s00281-009-0174-3 19707762

[144] Herbrand H Bernhardt G Förster R Pabst O Dynamics and function of solitary intestinal lymphoid tissue. Crit Rev Immunol (2008) 28:1–13 10.1615/CritRevImmunol.v28.i1.10 18298381

[145] Rescigno M Urbano M Valzasina B Francolini M Rotta G Bonasio R Dendritic cells express tight junction proteins and penetrate gut epithelial monolayers to sample bacteria. Nat Immunol (2001) 2:361–7 10.1038/86373 11276208

[146] Lelouard H Fallet M de Bovis B Méresse S Gorvel JP Peyer’s patch dendrites cells sample antigens by extending dendrites through M cell-specific transcellular pores. Gastroenterology (2012) 142:592–601.e3 10.1053/j.gastro.2011.11.039 22155637

[147] Knoop KA Miller MJ Newberry RD Transepithelial antigen delivery in the small intestine: different paths, different outcomes. Curr Opin Gastroenterol (2013) 29:112–8 10.1097/MOG.0b013e32835cf1cd 23380572 PMC3697126

